# Neoadjuvant Accelerated Methotrexate, Vinblastine, Doxorubicin, and Cisplatin Chemotherapy for Muscle-Invasive Urothelial Cancer: Large, Single-Center Analysis of Consecutive Patients’ Data

**DOI:** 10.3390/cancers17020258

**Published:** 2025-01-14

**Authors:** Łukasz Kwinta, Kamil Konopka, Krzysztof Okoń, Mateusz Łobacz, Piotr Chłosta, Przemysław Dudek, Anna Buda-Nowak, Paweł Potocki, Piotr J. Wysocki

**Affiliations:** 1 Oncology Department, Faculty of Medicine, Jagiellonian University Medical College, 31-501 Krakow, Poland; lukasz.kwinta@uj.edu.pl (Ł.K.);; 2 Clinical Department of Oncology, University Hospital in Krakow, 31-501 Kraków, Poland; 3 Pathomorphology Department, Faculty of Medicine, Jagiellonian University Medical College, 31-531 Krakow, Poland; 4 Department of Pathomorphology, University Hospital in Krakow, 30-688 Kraków, Poland; 5 Urology Department, Faculty of Medicine, Jagiellonian University Medical College, 30-688 Krakow, Poland; 6 Clinical Department of Urology and Oncological Urology, University Hospital in Krakow, 30-688 Kraków, Poland

**Keywords:** bladder cancer, urothelial cancer, neoadjuvant chemotherapy, aMVAC

## Abstract

**Background/Objectives**: Bladder cancer is a significant clinical problem with approximately 500,000 new cases worldwide annually. In approximately 25% of cases, disease is diagnosed at a stage of invasion of the muscle layer of the bladder. The current standard approach in this disease is preoperative chemotherapy followed by radical cystectomy. Dose-dense MVAC (ddMVAC), a two-day chemotherapy regimen, is the reference treatment protocol in this setting. The presented study evaluated the effectiveness and safety of accelerated MVAC (aMVAC) chemotherapy—a one-day regimen given before the resection of the bladder due to muscle-invasive disease. **Methods**: A retrospective analysis included 119 consecutive patients diagnosed with urothelial muscle-invasive bladder cancer (MIBC) who underwent preoperative chemotherapy with the aMVAC regimen. The planned treatment included 4–6 cycles of preoperative chemotherapy. The analysis of the degree of histopathological response to treatment was based on the three-grade TRG (tumor regression grade) classification. **Results**: A complete pathological response (TRG1) was observed in 44 patients (36.7%), and a major pathologic response (<ypT2) was achieved in 58 patients (48.7%). A reduction in the cisplatin dose was associated with a statistically significant decrease in the chance of achieving complete pathologic responses (46.1% vs. 10%, RR for TRG1 = 0.69, *p* = 0.00118). Patients who received at least 4 cycles (compared to ≤3 cycles) of neoadjuvant chemotherapy had a significantly higher chance of achieving a pathological response (partial or complete) to treatment (78.1% vs. 52.2%, RR 0.68, *p* = 0.0374). Administration of at least five cycles of chemotherapy was associated (compared to four cycles) with a significantly higher likelihood of achieving a complete pathological response (63.2% vs. 33.8%, RR = 1.71, *p* = 0.0221). The vast majority of adverse events were in grades 1 and 2, according to CTCAE version 5.0. Only five patients experienced grade 3–4 toxicities. The most common adverse event was anemia, which occurred in 66.3% of patients. **Conclusions**: Our real-world data analysis confirms the activity, safety, and feasibility of the aMVAC regimen as neoadjuvant chemotherapy in patients with urothelial MIBC.

## Simple Summary

Bladder cancer is a significant clinical problem with approximately 500,000 new cases worldwide annually. Muscle-invasive urothelial carcinoma represents a potentially fatal disease. Radical cystectomy preceded by neoadjuvant chemotherapy is the primary treatment modality for this condition. The present study evaluated the effectiveness and safety of accelerated MVAC (aMVAC) chemotherapy used before the resection of the bladder due to muscle-invasive disease. We demonstrated that preoperative aMVAC chemotherapy, a one-day regimen, is an effective and safe treatment option in this setting.

## 1. Introduction

Bladder cancer accounts for 3% of all malignant neoplasms, making it the 10th most common cancer (6th among men and 17th among women) [1]. Globally, approximately 500,000 new cases of this cancer were reported in 2020. At the same time, this disease caused 2.1% of cancer-related deaths, translating into around 210,000 deaths worldwide. The incidence of bladder cancer increases with age, with a median age at diagnosis of 73 years. Approximately 98% of cases occur after the age of 45 [2].

The key cause of bladder cancer development is carcinogenesis induced by the presence of xenobiotics in urine and their mutagenic effect on the urothelial mucosa [3]. The primary risk factor responsible for bladder cancer development is smoking (it is estimated to account for over 50% of cases) [4]. Other significant risk factors include occupational exposure to chemical carcinogens, particularly aromatic amines [5].

The most common histological type of bladder cancer is urothelial carcinoma (UC), which originates from the transitional epithelium lining the urinary tract (accounting for approximately 95% of cases). Among urothelial cancers, 15–25% include subtypes (e.g., nested, micropapillary, plasmacytoid, sarcomatoid, or poorly differentiated urothelial carcinoma), as well as tumors with divergent differentiation (e.g., squamous, glandular, or trophoblastic differentiation) [6].

Due to the uncommon nature of these histopathological variants, there are no distinct guidelines for their systemic or local treatments. Nevertheless, the progression and response to oncological therapies of rare urothelial cancer variants may vary from those of classical urothelial carcinoma.

Most bladder cancers (approximately 75%) are diagnosed at a noninvasive stage (limited to the mucosa or submucosal connective tissue without muscle layer invasion) [7]. In the case of non-muscle invasive urothelial cancer (NMIBC), the treatment of choice is local therapy: transurethral resection of bladder tumor (TURBT) complemented, if indicated, by intravesical therapy (BCG immunotherapy or chemotherapy).

The primary treatment for muscle-invasive bladder cancer (MIBC) is radical cystectomy. Given the unsatisfactory outcomes of surgical treatment alone, attempts have been made to improve patients’ outcomes with the use of perioperative systemic therapy. The current standard approach in this context is preoperative chemotherapy. The GETUG-AFU V05 VESPER (VESPER) phase III trial (NCT01812369) has defined the reference treatment protocol for neoadjuvant chemotherapy in MIBC [8]. This study compared the effectiveness of the cisplatin and gemcitabine combination (PG) regimen with ddMVAC (dose-dense MVAC: methotrexate, vinblastine, doxorubicin, cisplatin), demonstrating the significant superiority of the ddMVAC regimen. Results of a recently published phase III study NIAGARA (NCT03732677) demonstrated that the addition of immunotherapy (durvalumab) increases the activity of neoadjuvant chemotherapy, but the chemotherapy backbone was based on the suboptimal (PG) regimen [9]. Therefore, the results of NIAGARA, albeit intriguing, have not dethroned ddMVAC as the neoadjuvant regimen of choice in MIBC. However, the ddMVAC regimen poses a particular logistical challenge because this regimen must be administered over two consecutive days (methotrexate on day 1; vinblastine, doxorubicin, and cisplatin on day 2), which often complicates patient compliance, requiring either overnight hospitalization or outpatient visits on two consecutive days.

Our analysis sought to assess the effectiveness and safety of a one-day ddMVAC modification, known as the accelerated MVAC (aMVAC) regimen. This approach offers a distinct opportunity to reduce the strain on patients, staff, and healthcare facilities while maintaining the activity of a classical two-day ddMVAC regimen by allowing patients to receive complete chemotherapy on day 1 within 5 h without a need for a next-day visit.

## 2. Materials and Methods

The retrospective analysis included 119 patients diagnosed with urothelial MIBC (diagnosed based on TURBT) who underwent preoperative chemotherapy with the aMVAC regimen. The patients were treated between 2016 and 2024 at the Clinical Department of Oncology of the University Hospital in Krakow.

The preoperative chemotherapy regimen (aMVAC) consisted of methotrexate at 30 mg/m^2^, vinblastine at 3 mg/m^2^, doxorubicin at 30 mg/m^2^, and cisplatin at 70 mg/m^2^ administered on day one of the biweekly cycles. Due to the >20% risk of febrile neutropenia, treatment was conducted with pegfilgrastim support as primary prophylaxis, administered subcutaneously at a dose of 6 mg 24–48 h after the completion of chemotherapy.

The planned treatment included 4–6 cycles of preoperative chemotherapy. In the case of intolerance or urgent clinical conditions requiring expedited surgery (e.g., bladder bleeding), systemic treatment was shortened. Following the completion of systemic therapy, patients underwent surgical treatment (radical cystectomy) at the Clinical Department of Urology and Oncological Urology of the University Hospital in Krakow.

Before the initiation of treatment, all patients underwent staging, including a computed tomography (CT) scan of the chest, abdomen, and pelvis with intravenous contrast. Due to the use of doxorubicin, baseline echocardiography was performed for all patients.

Neoadjuvant chemotherapy was given to patients diagnosed with MIBC cT2-cT4a and cN0-cN3 without distant metastases. Patients who qualified for therapy had appropriate renal function with a GFR > 60 mL/min and a left ventricular ejection fraction of at least 50% [Table 1].

A dedicated, experienced pathologist at the Department of Pathomorphology of the University Hospital in Krakow conducted the histopathological examination of surgical specimens. The analysis of the degree of histopathological response to treatment was based on the three-grade TRG (tumor regression grade) classification developed initially by Fleischmann A et al. [10]. In the Fleischmann scale, TRG1 represents a complete response: no tumor cells and extensive fibrosis in the tumor bed; TRG2 represents a strong (partial) response: dominant fibrosis in the tumor bed, with residual tumor tissue comprising <50% of this area; TRG3 represents a poor response or no response: dominant tumor tissue outweighing fibrosis in the tumor bed (≥50% of the area occupied by tumor cells) or no regressive changes (indicating lack of tumor regression).

At the Department of Pathomorphology of the University Hospital in Krakow, this scale was applied with a modification: changes not exceeding the basement membrane of the mucosa (pTa and pTis) were classified as TRG1.

The chi-square test was used to analyze categorical variables, and a one-way ANOVA was performed to analyze continuous variables. Unconditional maximum likelihood (Wald) was used to calculate risk ratios for categorical variables.

The analyses were performed in R software (The R Foundation for Statistical Computing, version 4.3.1). All tests were two-sided, and statistical significance was defined as *p* < 0.05. No correction was applied for multiple statistical tests due to this study’s exploratory nature.

## 3. Results

### 3.1. Pathological Responses in General Population

**Figure 1:**
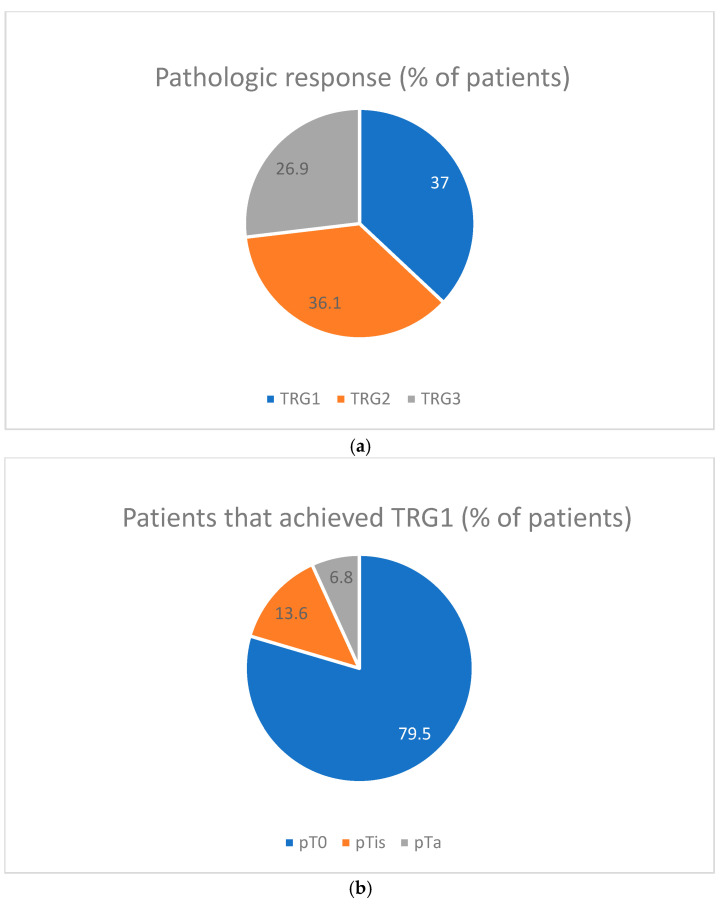
Pathological response: (**a**) pathological response in general population (% of patients); (**b**) distribution of pathologic response within TRG1 group (% of patients).

A complete response (TRG1) was observed in 44 patients (36.7%), including pT0 in 35 patients (29.4%). Downstaging to non-muscle invasive cancer (<ypT2) was achieved in 58 patients (48.7%). Partial pathologic response (TRG2) was noted in 43 patients (36.1%), while no response (TRG3) was observed in 32 patients (26.9%) [Figure 1].

**Figure 2:**
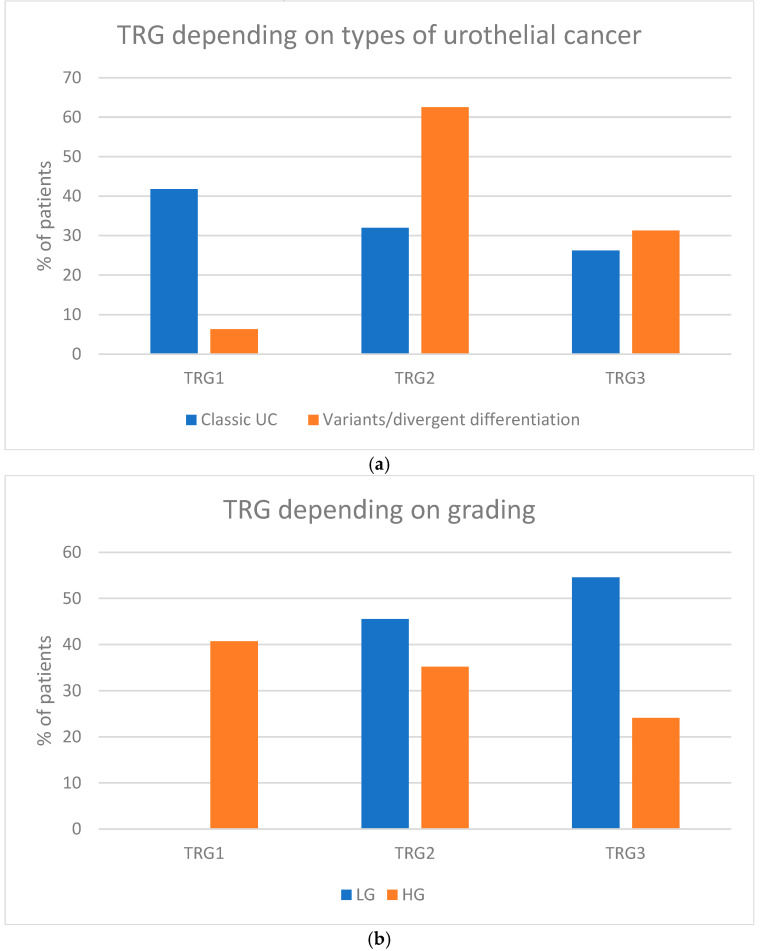
Pathological response depending on histopathological features (% of patients): (**a**) types of urothelial cancer (classic UC vs. variants of UC/UC with divergent differentiation); (**b**) grading of UC (low grade vs. high grade).

The diagnosis of classic urothelial carcinoma was associated with a higher likelihood of achieving a complete pathologic response (41.8%) compared to urothelial carcinoma variants, where TRG1 was achieved in only one patient (6.3%) [Figure 2a]. Similarly, the presence of high-grade (HG) cancer was a predictive factor for achieving a complete pathologic response associated with 40.7% of the TRG1 rate. In contrast, none of the patients with low-grade (LG) cancer achieved TRG1 [Figure 2b].

**Figure 3:**
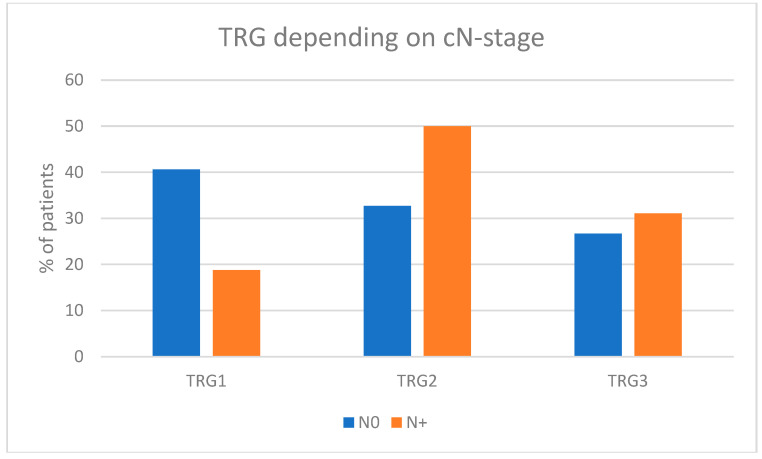
Pathological response depending on regional lymph nodes’ involvement (CT staging) prior to neoadjuvant chemotherapy (% of patients).

The baseline disease stage correlated with the final effect of preoperative chemotherapy. Advanced primary tumor stage cT4a and the involvement of pelvic lymph nodes were associated with a lower likelihood of achieving a complete response. The TRG1 rate in cT4a patients was 9.1% compared to 40.4% in cT2 and 40.7% in cT3 patients. The TRG1 rate in cN+ patients was 18.8% compared to 40.6% in patients without regional lymph node involvement [Table 2; Figure 3].

### 3.2. The Impact of Dose Reduction and Treatment Delays

**Figure 4:**
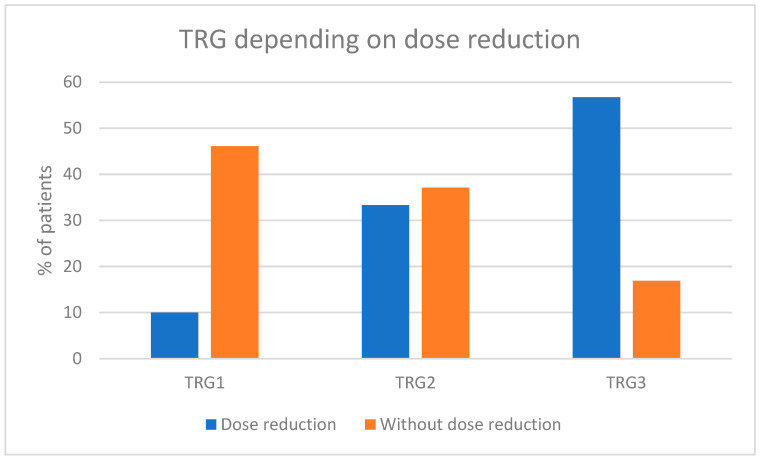
The impact of cisplatin dose reduction on the pathological response.

A reduction in the cisplatin dose was associated with a statistically significant decrease in the chance of achieving complete pathologic responses. The rate of TRG1 in patients without cisplatin dose reduction was 46.1% compared to 10% in patients who required cisplatin dose reduction (*p* = 0.00118, RR for TRG1 = 0.69) [Figure 4].

Conversely, treatment delays were not associated with diminished activity of neoadjuvant chemotherapy. The TRG1 rate in patients treated with planned intensity was 37.7% compared to 35.7% in patients who experienced treatment delays [Table 2].

### 3.3. The Impact of the Number of Chemotherapy Cycles

**Figure 5:**
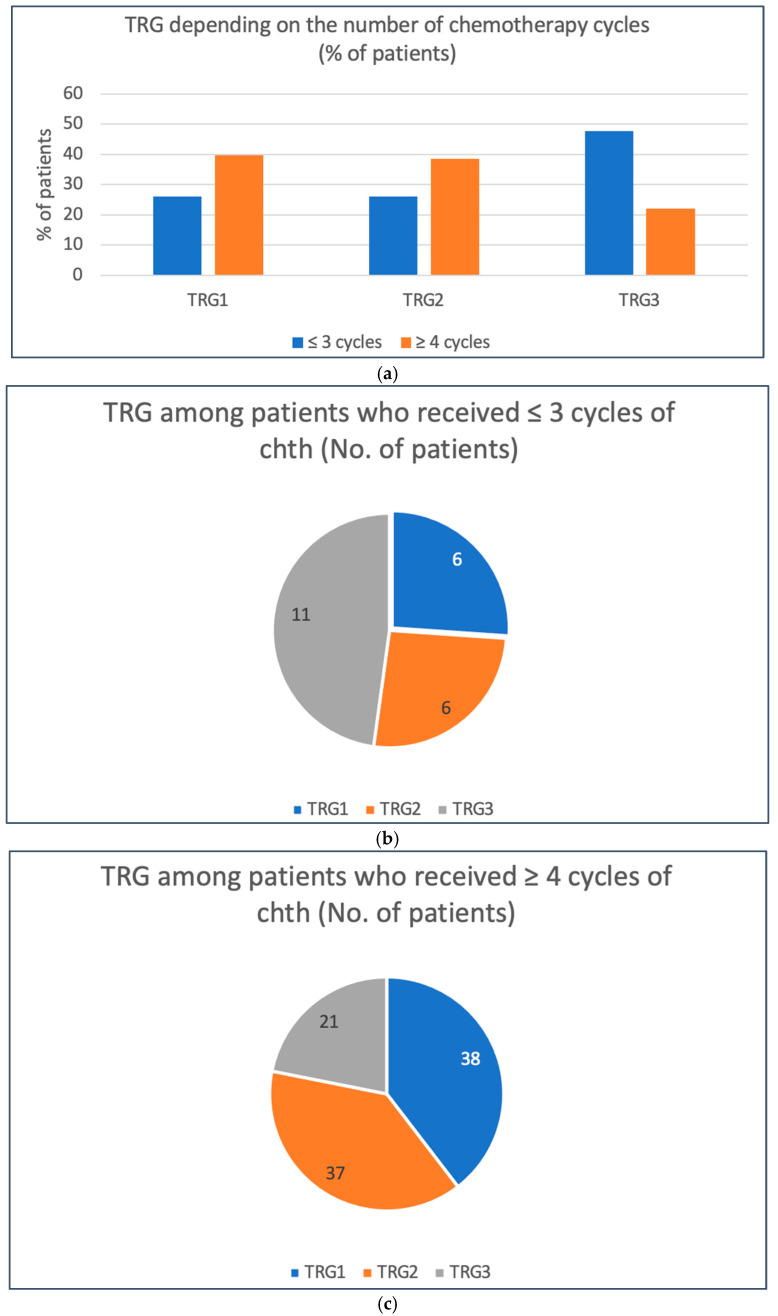
Treatment responses depending on the number of chemotherapy cycles (≤3 vs. ≥ 4cycles): (**a**) comparison between two groups (% of patients); (**b**) TRG among patients who received ≤ 3 cycles of treatment (number of patients); (**c**) TRG among patients who received ≥4 cycles of treatment (number of patients).

**Figure 6:**
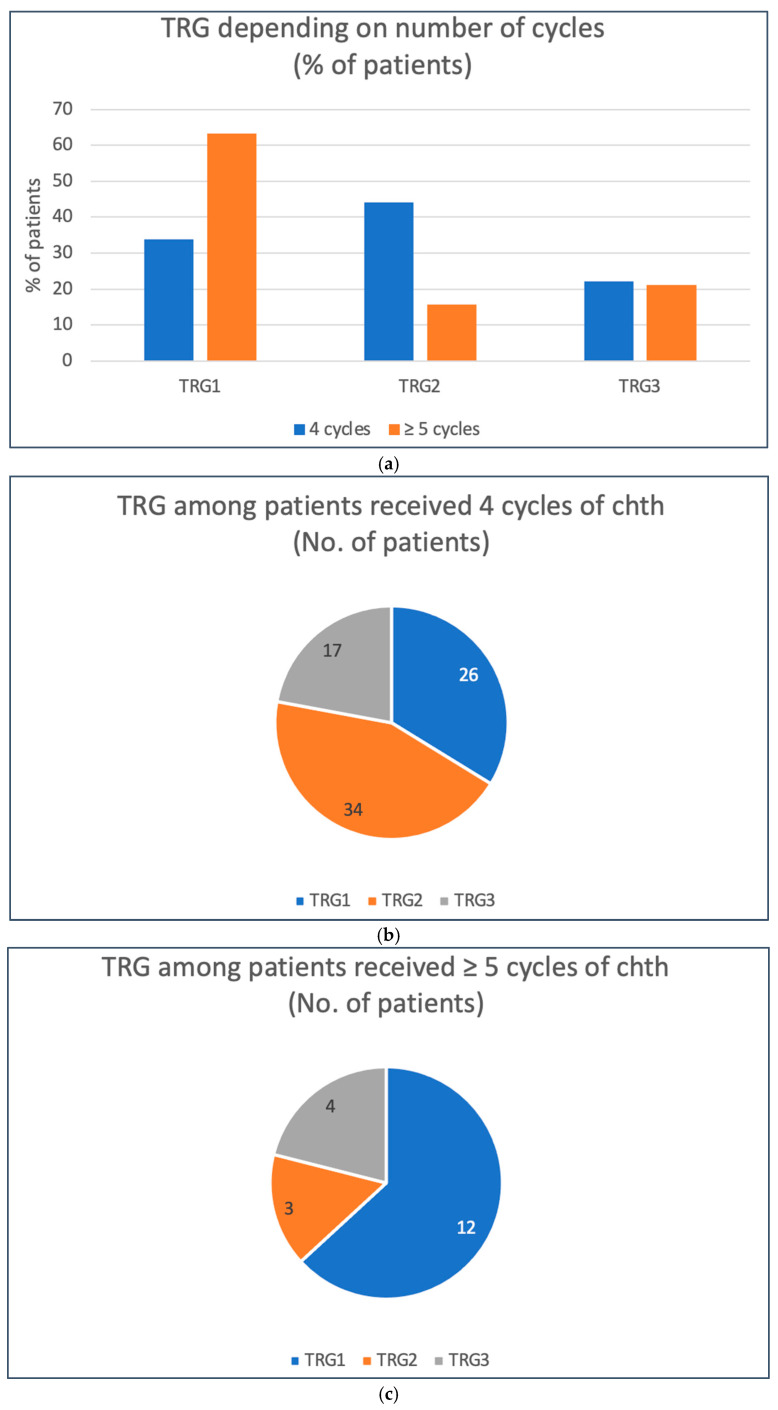
Treatment responses depending on the number of chemotherapy cycles (4 vs. ≥5 cycles): (**a**) comparison between two groups (% of patients); (**b**) TRG among patients who received 4 cycles of treatment (number of patients); (**c**) TRG among patients who received ≥5 cycles of treatment (number of patients).

Patients who received at least four cycles of neoadjuvant chemotherapy had a significantly higher chance of achieving a pathological response to treatment (partial (TRG2) or complete (TRG1)). In this group, a response was achieved in 78.1% of patients compared to 52.2% of patients who received up to three cycles of therapy (RR 0.68, *p* = 0.0366, unconditional maximum likelihood (Wald) test) [Table 3; Figure 5]. On the other hand, administration of at least five cycles of chemotherapy was associated (compared to four cycles of treatment) with a significantly higher likelihood of achieving a complete pathological response (63.2% vs. 33.8%, respectively, RR = 1.71, *p* = 0.0221, unconditional maximum likelihood (Wald) test) [Table 4; Figure 6].

### 3.4. The Impact of Delayed Surgical Treatment

The time from the completion of preoperative chemotherapy to the surgical procedure did not influence the complete pathological response rate (mean of 49.9 days for TRG1 vs. 51.3 days for the TRG2+3 group, *p* = 0.732). However, this time was significant regarding the percentage of patients who showed any pathological response to treatment. A lack of response on a pathological assessment was observed significantly more often in patients with delayed surgery (mean time of chemotherapy-free period—58.3 days) compared to in those who underwent cystectomy within a shorter time after chemotherapy (mean 47.8 days) (*p* = 0.0155) [Table 5].

### 3.5. Neoadjuvant Chemotherapy Safety

The vast majority of reported adverse events were of grades 1 and 2 according to CTCAE (Common Terminology Criteria for Adverse Events) version 5.0. Hematological toxicities were the most common among them. The most frequent was anemia, which occurred in 66.3% of patients, but in the majority (52.2%) it was a grade 1 adverse event. Only one patient experienced G3 anemia. Thrombocytopenia affected 23.5% of patients, with no episodes reported in grades 3–4. Due to primary prophylaxis of febrile neutropenia with pegfilgrastim, neutropenia and febrile neutropenia were relatively rare (neutropenia in 17.6% of patients, with G3 events in three patients, and no G4 episodes); the only grade 4 adverse event was febrile neutropenia in one patient. Renal toxicity and hepatotoxicity did not exceed grade 2 and affected 18.5% and 13.2% of patients, respectively [Table 6].

## 4. Discussion

Neoadjuvant chemotherapy represents a standard approach in patients with urothelial MIBC. However, there is still some discussion about whether the neoadjuvant approach is indeed better than adjuvant systemic treatment. Although some analyses indicate that cancer-specific survival is similar between neoadjuvant and adjuvant approaches, the overall survival data still support the preoperative strategies [11,12]. Adjuvant systemic treatment in large prospective studies could not significantly improve overall survival despite the significant improvement of event-free survival [13]. Still, a recent meta-analysis demonstrated that adjuvant chemotherapy may indeed be associated with improved overall survival [14]. All current guidelines recommend administering dose-intensive chemotherapy regimens like ddMVAC, ddGC (dose-dense GC: gemcitabine, cisplatin), or aMVAC [15,16,17]. However, the most robust data support ddMVAC, representing the only regimen to be evaluated within a phase III clinical study (GETUG-AFU-VESPER) [8]. The accelerated MVAC regimen, which is much more feasible from logistical and patient preference points of view, is supported by much weaker data. A phase II study evaluated accelerated MVAC (aMVAC) in 44 patients with urothelial MIBC [18]. Complete pathological response (pT0) to three cycles of aMVAC was observed in 38% of patients, and another 8% achieved pTis or pTa. Overall, 54% of patients who received aMVAC demonstrated downstaging to non-muscle-invasive disease <ypT2. The treatment was well tolerated, with 82% of patients experiencing only grade 1–2 treatment-related adverse events and no grade 3–4 renal toxicities. Our analysis confirms the high activity of aMVAC, albeit with a lower rate of ypT0 (29%) and <ypT2 downstaging (49%). The slightly lower rate of response to aMVAC in our study may be associated with a trial non-selected patient population reflecting a real-life clinical practice and with slight differences in the initial clinical stage of the disease as follows: cT2 40% vs. 36%, cT3 50% vs. 43%, cT4 9% vs. 14%, and N+ 14% vs. 7% for our and Plimack’s analyses, respectively. Compared to Plimack’s study, the GETUG-AFU-VESPER study, which evaluated six cycles of neoadjuvant ddMVAC, demonstrated even higher response rates (ypT0–42%, <ypT2–63%). However, the percentage of patients with initially muscle-confined MIBC (cT2) in the VESPER study was 90% compared to only 40% in our analysis.

Complete pathological response (pCR) is a strong prognostic factor for long-term outcomes in various cancers, including MIBC [19,20]. It provides early confirmation of the activity of neoadjuvant chemotherapy and allows for the optimization of postoperative systemic treatment. The VESPER study demonstrated that the pCR rate depends on the activity of the chemotherapy regimen (ddMVAC was superior to GC) [8]. Cisplatin is a critically important agent used for the treatment of urothelial cancers both in curative and palliative settings [21,22]. Our analysis revealed that any reduction in the cisplatin dose was linked to a lower likelihood of achieving a pathological complete response (pCR). This finding suggests that maintaining the optimal cisplatin dosage should be prioritized, even if adjustments are made to other components of the aMVAC regimen. Our analysis additionally shows that the pCR rate is associated with administering a particular number of aMVAC cycles. At least five cycles of aMVAC were associated with the highest probability of pCR and thus with the lowest risk of disease regrowth upon delayed surgery.

One of the important drawbacks of our analysis is the lack of comprehensive survival data of our patients. Many MIBC patients who were referred to our center for surgery and underwent neoadjuvant chemotherapy were lost to follow-up at our hospital due to being taken care of by their primary urologists. Therefore, we were unable to present data regarding event-free or cancer-specific survival. However, we continue to collect data on overall survival, which, as for now, are not mature and do not provide any reliable information on patients’ outcomes.

The optimal number of neoadjuvant chemotherapy cycles in MIBC is not well established. On the one hand, older age, diminished organ reserves, and comorbidities characteristic of patients with MIBC represent risk factors for increased toxicity. On the other hand, even though the median age of patients in the VESPER trial was 63 years (58–68), the majority of them (61%) were capable of receiving six cycles of neoadjuvant chemotherapy. There were three (1%) treatment-related deaths in the VESPER study, and grade 3–4 toxicities (mainly hematological) occurred in 52% of patients. A significant cardiovascular or renal impairment incidence was relatively low (each occurring in approximately 6% of patients) [23]. In our analysis, the median number of cycles was 4, with 81% of patients receiving ≥4 cycles and 16% ≥5 cycles of aMVAC. The treatment was also well tolerated in our real-life population, with G3 toxicities occurring in three patients and G4 in one patient.

Our analysis demonstrated that the number of chemotherapy cycles is an important predictive factor for the benefit of neoadjuvant therapy. While receiving less than four cycles was significantly correlated with an increased risk of neoadjuvant chemotherapy failure (TRG3), receiving less than five cycles significantly diminished the chance for a complete pathological response. These results clearly show that the minimal number of chemotherapy cycles that benefit patients is four and that fragile patients in suboptimal performance status who are at risk of not completing at least four cycles of chemotherapy should be considered for upfront cystectomy.

Prolonged treatment-related toxicity and logistical reasons may delay the moment of cystectomy in patients who have completed neoadjuvant chemotherapy. There is no consensus on the optimal duration of the post-chemotherapy period, which would allow patients to recover from chemotherapy sequelae and to prepare for the surgical procedure optimally. The median delay of surgery from the last course of chemotherapy in our analysis was 48 (17 to 122) days, which was virtually the same as in the VESPER study—a median of 48 days (95% range—27 to 97 days). While delaying surgery had no impact on the probability of the complete pathological response, it significantly increased the risk of detection of chemotherapy unresponsiveness (TRG3). This observation indicates that delayed surgery negatively impacts patients who are not achieving pCR, in whom the delay allows for cancer cell recovery, regrowth, and possibly progression, which could worsen patient outcomes.

Our analysis demonstrated that patients with organ-confined disease had a higher chance of complete response (TRG1) than those without. However, available data suggest that even though neoadjuvant chemotherapy is associated with a lower probability of complete response in patients with non-organ-confined disease, the scale of improvement of long-term outcomes with the use of preoperative treatment is larger in non-organ-confined than in organ-confined MIBC patients [24]. However, the involvement of locoregional lymph nodes represents a detrimental risk factor associated with significantly worse overall survival and decreased benefit from neoadjuvant chemotherapy than in patients with clinically negative lymph nodes [25].

The population of patients in our analysis was very homogenous for race and ethnicity since 100% of patients were Caucasian. Available retrospective studies have demonstrated that Caucasian MIBC patients are most likely to benefit from various radical approaches (neoadjuvant chemotherapy before radical cystectomy or trimodal therapy) compared especially to African Americans [26,27].

Our analysis has not involved patients with UTUC, because previous guidelines on the treatment of urothelial cancer have not supported the use of neoadjuvant chemotherapy in such patients and endorsed primary nephroureterectomy followed by adjuvant chemotherapy. The use of adjuvant treatment was supported by a phase III clinical trial (POUT, NCT01993979), which demonstrated significant improvement in event-free survival following gemcitabine and cisplatin/carboplatin combination [28]. However, some recent guidelines endorse the use of neoadjuvant chemotherapy in UTUC patients with N+ disease. These guidelines are based on retrospective but robust data demonstrating significantly better outcomes in UTUC N+ patients who received standard (as in MIBC) neoadjuvant chemotherapy [29,30,31].

## 5. Conclusions

Our real-world data analysis clearly supports available data on the activity, safety, and feasibility of the aMVAC regimen used in neoadjuvant settings in patients with urothelial MIBC. Patients should receive at least four cycles of neoadjuvant chemotherapy, with every effort to give at least five to maximize the chance of a complete pathological response.

## Figures and Tables

**Table 1:** Patients’ characteristics.

Overall		119 (100%)
Race	Caucasian	119 (100%)
Ethnicity	Hispanic or Latino	0 (0%)
Sex	Male	95 (79.8%)
	Female	24 (20.2%)
Age	Median (min–max) [years]	65 (34–80)
Family history	Incidences of MIBC in family members	0 (0%)
Histopathologic type	Classic urothelial cancer (UC)	103 (86.6%)
	Histologic subtypes of UC	6 (5.0%)
	UC with divergent differentiation	10 (8.4%)
Grading	High grade	108 (90.8%)
	Low grade	11 (9.2%)
Primary clinical stage—T	T2	47 (39.5%)
	T3	59 (49.6%)
	T4a	11 (9.2%)
	Unknown	2 (1.7%)
Primary clinical stage—N	N0	101 (84.9%)
	N1	7 (5.9%)
	N2	4 (3.4%)
	N3	5 (4.2%)
	Unknown	2 (1.7%)
History of UTUC (Upper Tract UC)		0 (0%)
Number of cycles of chemotherapy	Median (min–max)	4 (1–6)
	1	2 (1.7%)
	2	3 (2.5%)
	3	18 (15.1%)
	4	77 (64.7%)
	5	10 (8.4%)
	6	9 (7.6%)
Dose reductions	Yes	30 (25.2%)
	No	89 (74.8%)
Dose delays	Yes	42 (35.3%)
	No	77 (64.7%)

**Table 2:** Response for treatment in respect to clinical and histopathologic features.

		n (%)
TRG	1	44 (37.0%)
	2	43 (36.1%)
	3	32 (26.9%)
TRG1	ypT0 ypN0	35 (29.4)
	ypTis and ypTa ypN0	9 (7.6)
ypT1 ypN0		14 (11.8)
Major response (<ypT2 ypN0)		58 (48.7)
Classic urothelial cancer—TRG (n = 103)	1	43 (41.8)
	2	33 (32.0)
	3	27 (26.2)
Variants/divergent differentiation—TRG (n = 16)	1	1 (6.3)
	2	10 (62.5)
	3	5 (31.3)
High grade (HG)—TRG (n = 108)	1	44 (40.7)
	2	38 (35.2)
	3	26 (24.1)
Low grade (LG)—TRG (n = 11)	1	0 (0.0)
	2	5 (45.5)
	3	6 (54.6)
Primary cT2—TRG (n = 47)	1	19 (40.4)
	2	18 (38.3)
	3	10 (21.3)
Primary cT3—TRG (n = 59)	1	24 (40.7)
	2	18 (30.5)
	3	17 (28.8)
Primary cT4—TRG (n = 11)	1	1 (9.1)
	2	5 (45.5)
	3	5 (45.5)
Primary cN0—TRG (n = 101)	1	41 (40.6)
	2	33 (32.7)
	3	27 (26.7)
Primary cN+—TRG (n = 16)	1	3 (18.8)
	2	8 (50.0)
	3	5 (31.3)
Dose reduction—TRG (n = 30)	1	3 (10.0)
	2	10 (33.3)
	3	17 (56.7)
Without dose reduction—TRG (n = 89)	1	41 (46.1)
	2	33 (37.1)
	3	15 (16.9)
Dose delays—TRG (n = 42)	1	15 (35.7)
	2	16 (38.1)
	3	11 (26.2)
Without dose delays—TRG (n = 77)	1	29 (37.7)
	2	27 (35.1)
	3	21 (27.3)

**Table 3:** Treatment responses depending on the number of chemotherapy cycles (≤3 vs. ≥4 cycles).

	≤3 Cycles (n = 23)	≥4 Cycles (n = 96)	Total (n = 119)	*p*-Value *
**TRG**				
1	6 (26.1%)	38 (39.6%)	44 (37.0%)	*p* = 0.649
2	6 (26.1%)	37 (38.5%)	43 (36.1%)	
3	11 (47.8%)	21 (21.9%)	32 (26.9%)	
**TRG 3 vs. 1 + 2**				
3	11 (47.8%)	21 (21.9%)	32 (26.9%)	*p* = 0.0374
1 + 2	12 (52.2%)	75 (78.1%)	87 (73.1%)	
**TRG 1 vs. 2 + 3**				
1	6 (26.1%)	38 (39.6%)	44 (37.0%)	*p* = 0.369
2 + 3	17 (73.9%)	58 (60,4%)	75 (63.0%)	

* *p*-Value calculated with chi-square test.

**Table 4:** Treatment responses depending on the number of chemotherapy cycles (4 vs. ≥5 cycles).

	4 Cycles (n = 77)	≥5 Cycles (n = 19)	Total (n = 96)	*p*-Value *
**TRG**				
1	26 (33.8%)	12 (63.2%)	38 (39.6%)	*p* = 0.041
2	34 (44.2%)	3 (15.8%)	37 (38.5%)	
3	17 (22.1%)	4 (21.1%)	21 (21.9%)	
**TRG 3 vs. 1 + 2**				
3	17 (22.1%)	4 (21.1%)	21 (21.9%)	*p* = 1.0
1 + 2	60 (77.9%)	15 (78.9%)	75 (78.1%)	
**TRG 1 vs. 2 + 3**				
1	26 (33.8%)	12 (63.2%)	38 (39.6%)	*p* = 0.0541
2 + 3	51 (66.2%)	7 (36.8%)	58 (60.4%)	

* *p*-Value calculated with chi-square test.

**Table 5:** The impact of time of surgery on the pathological response.

Time to Surgery (Days)	TRG 3 (n = 32)	TRG 1 + 2 (n = 87)	Total (n = 119)	*p*-Value *
Mean (SD)	58.3 (27.2)	47.8 (18.3)	50.8 (21.6)	0.0155
Median [min, max]	51.5 [21.0–122.0]	45.0 [17.0–121.0]	48.0 [17.0–122.0]	
**Time to Surgery (Days)**	**TRG 1 (n = 44)**	**TRG 2 + 3 (n = 75)**	**Total (n = 119)**	***p*-Value**
Mean (SD)	49.9 (18.3)	51.3 (23.4)	50.8 (21.6)	0.732
Median [min, max]	49.0 [20.0–121.0]	47.0 [17.0–122.0]	48.0 [17.0–122.0]	

* *p*-Value calculated with one-way ANOVA.

**Table 6:** Toxicities of treatment.

	Grade 1–2		Grade 3–4	
	n	%	N	%
Leukopenia	8	6.7	0	0
Neutropenia	18	15.5	3	2.5
Febrile neutropenia	-	-	1	0.8
Lymphopenia	4	3.4	0	0
Anemia	78	65.5	1	0.8
Thrombocytopenia	28	23.5	0	0
Nephrotoxicity (elevation of creatinine level)	22	18.5	0	0
Hepatotoxicity (elevation of ALT, AST, or bilirubin level)	16	13.4	0	0

## Data Availability

All data generated or analyzed during this study are included in this published article.

## Abbreviations

The following abbreviations are used in this manuscript:

aMVAC	Accelerated MVAC (methotrexate, vinblastine, doxorubicin, cisplatin)
ddMVAC	Dose-dense MVAC (methotrexate, vinblastine, doxorubicin, cisplatin)
Chth	Chemotherapy
CT	Computed tomography
CTCAE	Common Terminology Criteria for Adverse Events
HG	High grade
ddGC	Dose-dense GC (gemcitabine, cisplatin)
LG	Low grade
MIBC	Muscle-invasive bladder cancer
MVAC	Methotrexate, vinblastine, doxorubicin, cisplatin
NMIBC	Non-muscle invasive urothelial cancer
RR	Risk ratio
TRG	Tumor Regression Grade
TURBT	Transurethral resection of bladder tumor
UC	Urothelial cancer
UTUC	Upper Tract Urothelial Carcinoma
